# Biodegradation of textile dye Reactive Blue 160 by *Bacillus firmus* (Bacillaceae: Bacillales) and non-target toxicity screening of their degraded products

**DOI:** 10.1016/j.toxrep.2019.11.017

**Published:** 2019-12-04

**Authors:** Selvaraj Barathi, Chinnannan Karthik, Nadanasabapathi S, Indra Arulselvi Padikasan

**Affiliations:** a School of Environmental Science and Engineering, Sun Yat-Sen University, Guangzhou, People’s Republic of China; b College of Agriculture and Biotechnology, Institute of Crop Science, Zhejiang University, People’s Republic of China; c Shenzhen Institute, Health Science Center, People’s Republic of China; d Plant and Microbial Biotechnology Laboratory, Department of Biotechnology, School of Biosciences, Periyar University, Salem, 636 011, Tamil Nadu, India

**Keywords:** Reactive Blue 1600, *Bacillus firmus*, Biodegradation, Toxicity, Germination, Zebrafish embryos

## Abstract

The study was envisioned to evaluate the decolorization of Reactive Blue 160 (RB160) dye by using indigenous microbes. Contaminated soil from textile dye industry was collected from Noyyal river basin, Tamil Nadu, India. Potential dye degrading bacterial strain was recognized as *Bacillus firmus* by 16SrRNA gene sequencing analysis. RB160 dye (500 μg/ml) was effectively degraded by *B. firmus* and toxicological analyses were performed with RB160 and their degraded product. Phytotoxicity revealed that degraded product of RB160 into non-toxic nature by *B. firms*. Toxicity assays were carried out on root cells of *Allium cepa* and human skin cell line (CRL 1474). Toxicity analysis of *A. cepa* and cell line signifies that dye exerts toxic cause on the root cells and IC_50_ values of RB160 showed toxic to human skin cell lines, while degradation products of the dye are moderately less in toxic. Zebrafish embryo toxicity also evaluated by RB160 and degraded product on phenotypic deformation, survival, hatching and heartbeat rate. However, RB160 with concentration of 500 μg/ml decrease in the survival, hatching, heartbeat rate and induced phenotypic alterations. In which, degraded products exhibited significant development in zebrafish embryos as compared to dye. Based on the studies effects of RB160 and capability of *B. firmus* can effectively degrade RB160, and their degraded products were harmless to the environments and aquatic system.

## 1 Introduction

Aquatic environmental pollution caused by discharge of raw effluents from various manufacturing industries, is a major environmental problem in worldwide [1]. Among them textile industries used large amount of potable water, for example 200 l of potable water consumed to create 1 kg of textile products, finally untreated waste water discharged to aquatic environments [2]. These textile industry effluents contain more toxic compounds, which are deleterious to all living organisms. Particularly, reactive dye (10–15 %) is measured to be the most hazardous xenobiotics. The RB 160 shade is the most frequent effluent with the highest level of discharges emitted from the silk, nylon, and wool strand industries [3,4]. The presence of reactive dyes in water bodies prevents sunlight penetration and oxygen supply prominent to a natural demand for oxygen and creating an unfavorable impact on the water quality, and exhibit higher level of toxicity to plants and other aquatic animals [5,6]. Humans and animals are facing a higher risk of infections and other serious diseases like cancer and skin allergy, infection when the dosage of this mutagenic dyes increase [7].

Therefore, it is necessary to develop an effective, economical and environmental friendly method for exclusion of toxic dyes completely from the textile wastewater [8]. Several conventional methods are available for textile wastewater treatment such as physical, chemical [9,10] and some engineered techniques including adsorption [11], electrolysis [12], oxidation [13] and photo-ionization [14]. However, all these methods are having several constraints such as expensive, lesser capability, and assistant hazardous intermediates generations [[15], [16], [17]].

However, all these methods are having several constraints such as expensive, lesser capability, and assistant hazardous intermediates generations [16]. In this scenario, Microbial degradation of dyes has attracted a lot of attention due to its cost-effective and eco-friendly approach [18,19]. Several studies have been reported that microbes including bacteria [20], fungi [21] are able to decolorizing toxic dyes. Some microbial consortium has been accounted for productive dye expulsion and further deterioration of metabolites [22]. Moreover, treatment plants for toxic chemical degradations are generally cost effective so it can be overcome by biologically active plants and microbes in greater benefit. Some bio-rational plants or their derivatives such as *Allium cepa, Vicia faba*, and *Trades cantiapaludosa* are being used for several years for their mutagenic impacts on ionizing radiation [23]. Besides, synthetic mutagens are also well assessed their mutagenicity/clastogenicity against ecological toxins. Hence, It is recommended that the utilization of the micronuclei (MCN) assay in root of *A. cepa* and *V. faba*, quadruplicates of *Tradescantia*, and stamen-hair serves a reliable tool to assess genotoxicity [24].

Tirupur district in Tamil Nadu, India, is broadly recognized as textile city. More than 700 bleaching and dyeing industries are discharging 75,000 m^3^ of effluents per day into the Noyyal River and terrestrial areas in Tirupur [25]. However, there are no previous research on the dye treatment and utilizing their degraded products on indigenous microorganisms against human skin cell line. This study investigated the capability of the *B. firms* and their potential efficiency to degrade in higher concentration of RB160 dye present in soil collected from Tirupur located in the Noyyal river basin. The capability of *B. firmus* strain isolated from industrial dye polluted soil and we examined the decolorization and degradation of RB160. Besides, the phytotoxicity effects of RB160 and their *B. firms* degraded products on four important plant seed corn (*Zea mays*), green gram (*Vigna radiate*), black gram (*Vigna Mungo*) and groundnut (*Arachis hypogae*) are evaluated and this study involved to assess the genotoxic, cytotoxic effects on *Aillum cepa.* In addition, the value of using the human skin cell line (CRL-1474) and zebra fish embryos to give more information about the toxicity of RB160 dye and degraded products with them were studied.

## 2 Material and methods

### 2.1 Reagents

RB160 dye (Commercial grade) was procured from the local dyeing industry, Tirupur. Corn, green gram, groundnut, and black gram subjected to Phyto-toxicity studies were purchased from the local market and cell lines (CRL 1474) were obtained from Ramakrishna Hospital, Chennai, India.

### 2.2 Microorganisms and growth media

Bacterial strain *B. firmus* (GenBank accession numbers: KJ162242.1) was isolated from sediments of the wastewater treatment facility of a textile firm in the noyyal river basin of Komarapalayam (11.454501 and 77.696115), Tamil Nadu, India. Nutrient agar in the culture medium was used for isolating bacteria at an incubation temperature of 35 °C. Decolorization testing was executed with a mixture of RB 160 dye and the culture medium. The bacterial strain was inoculated to find decolorization of dyes at suitable conditions [26]. The isolated samples have the ability to decolorize six different reactive dyes, tested at the site of the dye manufacturing industry in Tirupur, Tamil Nadu, India. The isolated bacteria were characterized using 16SrRNA gene sequence study and with the assist of Bergey’s manual. The sequence was further confirmed by BLAST tool.

### 2.3 Decolorization and biodegradation process

The strain of *B. firmus* was used in toxicity studies was inoculated alone in NB medium and incubated at 30 °C for 24 h. Subsequently, cells were centrifuged at 6000 RPM for 10 min. Pellets were dissolved in the mineral salt medium (MSM). Cell density of 1.0 OD at 600 nm was made for decolorization experiment. The medium to inoculums ratio was continued at 50:1. MSM was prepared by adding RB160 with initial concentration of 500 ppm, after complete decolorization of RB160 was extracted with a same amount of ethyl acetate. The extracts were then dehydrated by vanishing; a tiny part of remaining residue was again dissolved in HPLC grade methanol and used for, phytotoxicity analysis in plant seeds, Cytotoxicity and genotoxicity analysis in *Allium cepa* and toxicity analysis on human skin cell line CRL-1474.

### 2.4 Toxicity testing of dye and its degraded products

#### 2.4.1 Phytotoxicity analysis in plants

Phytotoxicity study was done under laboratory temperature utilizing the germination of four different seeds: corn, green gram, groundnut, and black gram [27,28]. These were set up in both RB160 and degraded RB160. First, the seeds were soaked in 3 % hydrogen peroxide for 5 min and washed thrice with sterile distilled water. Fifty seeds of corn, black gram, groundnut, and green gram were placed into individual plates with 7 ml of RB160 treated and control. All the plates were then protect and developed in the greenhouse environment. Control tests utilizing distilled water rather than the RB160 dye arrangements were also maintained. The germination rates of the seeds were recorded after a week and the shoot and root lengths of the seeds were measured and documented. Comparative analysis and consolidated data were obtained for final description by performing the experiments in triplicate.

#### 2.4.2 Cytotoxicity and genotoxicity analysis in *Allium cepa*

For observation of the development of roots, petite bulbs of *A. cepa* of consistent size and shape were exposed to water. Three sets of bulbs were prepared. The first set of bulbs was exposed to the dye sample (500mgl^−1^ of RB160) for 48 h, the second set to metabolites of biodegraded dye (500mgl^−1^), and the third set was exposed to water as a control. After exposure to the respective chemicals and water, the bulbs were rinsed in running tap water and used for further genotoxicity and cytotoxicity studies [22].

### 2.5 Toxicity study

#### 2.5.1 Cell line medium and maintenance

Human skin cell line CRL-1474 was used to perform the cytotoxicity studies and cultures were maintained according to the Lamia ayed et al. [29].

#### 2.5.2 MTT cell proliferation assay

The MTT cell proliferation assay is a yellow color water-soluble tetrazolium salt assay where mitochondrial enzyme (succinate-dehydrogenase) in living cells cleaves the tetrazolium circle and changes the MTT to an inexplicable violet formazan. The quantity of formazan formed is straight relative to the numeral of viable cells in the test analysis. After 48 h of incubation, 15 μl of MTT (5 mg/ml) mixed with phosphate buffered saline (PBS) was additional to every well and incubated at 37 °C for 4 h. Then the medium with MTT was tapped off and the formazan salts shaped were solubilized in 100 μl of DMSO. The absorbance was precise at 570 nm by a micro-plate reader. The proportion of cell viability was calculated using the below formula (1)

Percentage of cell viability = [A] test**/** [A] control×100

#### 2.5.3 Comet assay toxicity test

The comet assay was done to evaluate the genotoxic potential of biodegraded products of the dye RB160. The constancy and consideration of this assay provide expedited prediction of the genotoxic capacity of the compounds by *in vivo* and *in vitro* monitoring of environmental pollutants. In this study, RB160 dye was exposed to human skin cell line (*CRL-1474*) to evaluate its genotoxicity. The nuclei were isolated from the CRL-1474 skin cell line and run in an electrophoresis unit. The nuclei were highly sensitive to the electric field due to the naked form and high nucleic acid content. The percentages of comet tail DNA, tail length (μm), and tail moment were measured [30].

#### 2.5.4 Zebrafish strains experiental design

Healthy embryos were collected from mature zebrafish and subjected for toxicity study. Collected embryos were arbitrarily separated into three groups (*n* = 10), the groups are; (i) control (embryo was maintained in the E3 medium), (ii) embryo treated with 250 μg/ml of RB 160 and (iii) embryo treated with a degraded product of RB160. Post treatment, embryos was observed for phenotypic deformation, hatching, survival and heart rate at specific time intervals under a light microscope (Olympus MLXi, Tokyo, Japan) connected to a digital camera.

### 2.6 Statistical analysis

All the experiments were performed in triplicates (n = 3) and the data was recorded. Seed germination rate was examined *via* the Kruskal-Wallis test and other experiments were analyzed by one-way ANOVA and means were evaluated with the Tukey’s test, using the SPSS software (version 20, SPSS Inc., www.spss.com).

## 3 Results and discussion

### 3.1 Phytotoxicity on plant seeds

The germination percentages of seeds were comparatively low with intact dye than the degraded product. The observed results demonstrate that the isolated *B. firmus* potentially decolorize the dye color as well as detoxify it. Consequently, toxicity of the dye was assessed before and after degradation. Table 1 illustrates that the seed germination percentage, length of radical, shoot, and the base (root) of the corn, green gram, black gram and groundnut. Comparatively, seed germination percentage was decreased with RB160 treatments compared to the extracted by-product and water (control). This result indicated that the metabolites produced after degradation of RB160 are less toxic in nature as compared to intact dye to the plants. The degraded metabolites were nontoxic to the experimented plants; subsequently the bacterial strain could be employed for remediation of textile waste *ex situ* or *in situ*. Generally, untreated dye effluents get dissolved in water bodies and when this water is utilized for agriculture, it affects fertility of fields and also causes health problems [31]. Therefore, it was the primary concern to estimate the textile dyes toxicity pre and post-treatment. Similarly, Parshetti et al. [32] reported that the comparative impacts of dyes and degradation products in relation to *Triticum aestivum* and *Phaseolus mungo* was performed and proved that germination was affected by intact malachite green dye.

**Table 1:** Phytotoxicity study of Reactive Blue 160 and its degradation product.

Parameter studied	Germination (%)*			Radical (cm)**			Shoot length (cm)**			Root length (cm)**		
Seeds	Water	R.B 160	Degradation product	Water	R.B 160	Degradation product	Water	R.B 160	Degradation product	Water	R.B 160	Degradation product
Corn	48.59 ± 0.98^a^	12.34 ± 2.11^bc^	46.30 ± 0.65^a^	3.16 ± 0.08^b^	ND	2.85 ± 0.25^ab^	8.6 ± 0.40^b^	ND	7.150 ± 0.05^b^	12.10 ± 0.80^a^	ND	12.50 ± 0.50^a^
Green gram	47.34 ± 1.54^ab^	15.76 ± 1.84^a^	43.90 ± 0.96^b^	4.50 ± 0.20^a^	ND	3.56 ± 0.20^a^	16.6 ± 0.23^a^	ND	16.50 ± 0.50^a^	7.80 ± 0.30^b^	ND	7.950 ± 0.05^b^
Ground nut	48.22 ± 1.09^a^	8.90 ± 1.09^c^	43.55 ± 1.31^b^	2.16 ± 0.08^bc^	ND	2.06 ± 0.43^ab^	4.66 ± 0.09^c^	ND	4 .133 ± 0.04^c^	7.467 ± 0.28^b^	ND	7.500 ± 0.25^b^
Black gram	46.98 ± 0.80^b^	14.34 ± 1.87^b^	45.12 ± 1.54^ab^	2.23 ± 0.07^bc^	ND	2.20 ± 0.09^ab^	5.46 ± 0.14^c^	ND	3.633 ± 0.27^c^	4.567 ± 0.29^c^	ND	3.800 ± 0.37^c^

**Note:** Values followed by different letters are significantly different at P < 0.05.

* Kruskal-Wallis – median and interquartile range.

** One-way ANOVA – mean ± standard error.

### 3.2 Toxicity test on *Alium cepa*

Evaluation of the environmental genotoxic effects of dyes and their break their products after biodegradation on plant populaces is highly needed as plants are of essential commercial value and are consumed by humans [33]. In this study, *A. cepa* were used for toxicological analysis of the dye and its degraded product. Genotoxicity effects of RB 160 dye was affirmed by a cytogenetic study of *A. cepa* root cells. Mitotic index (MI) plays a main role as a device to determine the cytotoxic effect of the dye. MI is an important optimizer to estimate the cytotoxicity studies through monitoring environment [34]. The genotoxic impact of RB 160 dye contrasted with its degradation product Supporting Information (SI). The mitotic index of *A. cepa* root cells were noticed at 500 ppm of RB 160 (14 ± 0.46) and their degraded product (10.9 ± 0.17), which are practically equivalent to that of control (11.5 ± 0.38). On the other side, the following various chromosomal aberrations was observed in mitotic index. The major chromosomal irregularity features in all dye treatments were chromosomal breaks, stickiness, micronuclei, anaphase bridges, and laggards. Based on the genotoxicity study results and the percentage of aberrant mitotic cells caused by the dyes were statistically significant compared to the control. Carita and Marin-Morales, [34] reported, when *A. cepa* roots were exposed to dye initiation in chromosome alteration with affect cell distribution process poorly were experiential to manufacturing effluents contaminated with azo dyes. The decreased mitotic index results indicated that the cytotoxic elements affect the mitotic cell division and reduced the plant growth [35].

### 3.3 Cytotoxic effects of RB160 dye and its degraded product of normal human skin cells CRL-1474

**Fig. 1:**
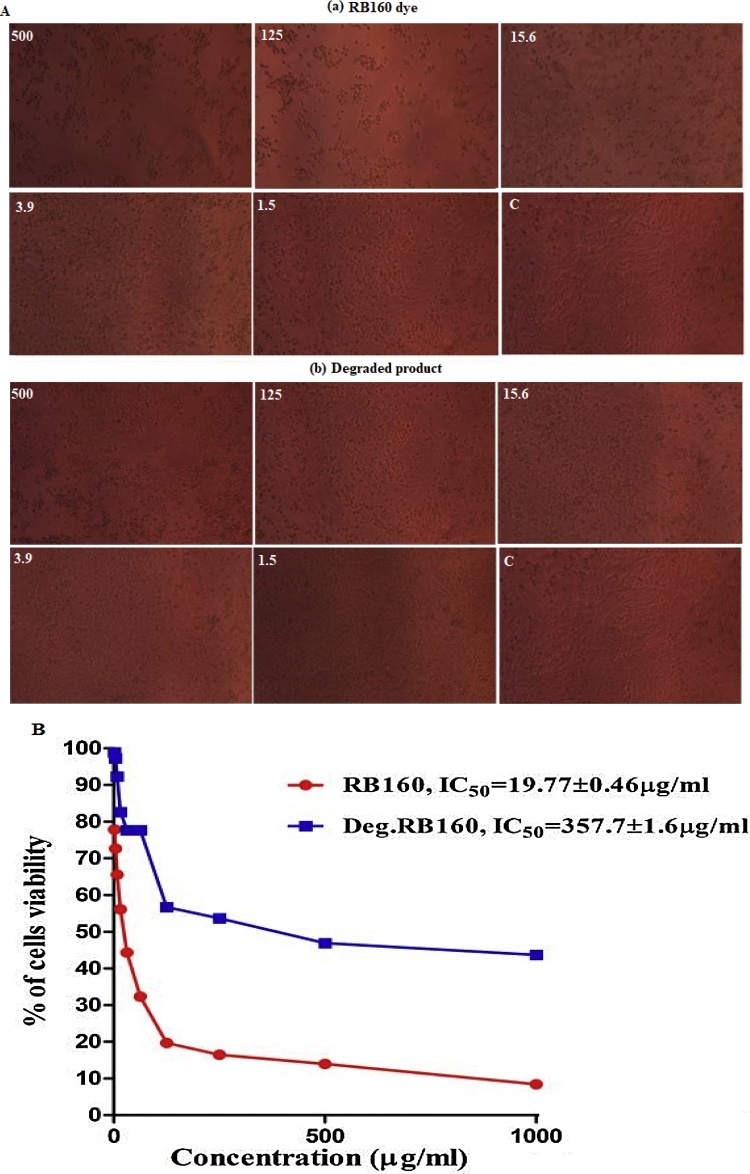
A) Photomicrograph represents a) MTT assays for RB160 in different concentration (500, 125, 15.62, 3.90, 1.59 μg/ml and control). b) The same concentration of degraded products in MTT assays and (B) Percentage of cell viability and IC_50_ values of RB 160 and degraded product.

Cytotoxicity effects of RB 160 dye and degraded products on normal human skin cells CRL-1474 was investigated by MTT assay and the results presented in Fig. 1a. The test was performed with different concentration of RB160 (500, 125, 15.62, 3.90, 1.59 μg/ml and control) and same range of their degraded products were used. The cytotoxic result of RB 160 treatment on the viability of skin cells with an IC_50_ range of 19.77 μg/ml at 24 h, whereas the degraded products showed the IC_50_ range is 357.7 μg/ml at 24 h. Hence, these IC_50_ concentrations were further used *in vitro* studies. This results show compared with native forms of RB160, degraded products show less toxic effect on normal human skin cells CRL-1474 (Fig. 1b). The IC_50_ value was increased 357.7 μg/ml after 24 h of degraded products treatments. However, very less IC_50_ values of RB 160 treatment was noticed 19.77 μg/ml at 24 h. These indicated that the degraded products were less toxic compared to the RB160 dye. Bafana et al. [36] used to evaluate the cytotoxicity of DR28 dyes at different concentration by Bacillus velezensis AB. Cytotoxicity was examined on HL-60 cell line utilizing by MTT test. Toxicity towards HL-60 cells expanded on the third day, trailed by a steady decrease up to fifteenth day (p < 0.01). Most of the studies have reported dyes showed large cytotoxicity effects compared to degraded products e.g mouse fibroblast cell line (L929 cell line) was used to investigate the effects of Reactive Red 141 and Reactive Red 2 dye and their degraded products by *Bacillus lentus* [37].

### 3.4 Genotoxic effects of RB160 dye and its degraded product on normal human skin cells CRL-1474

**Fig. 2:**
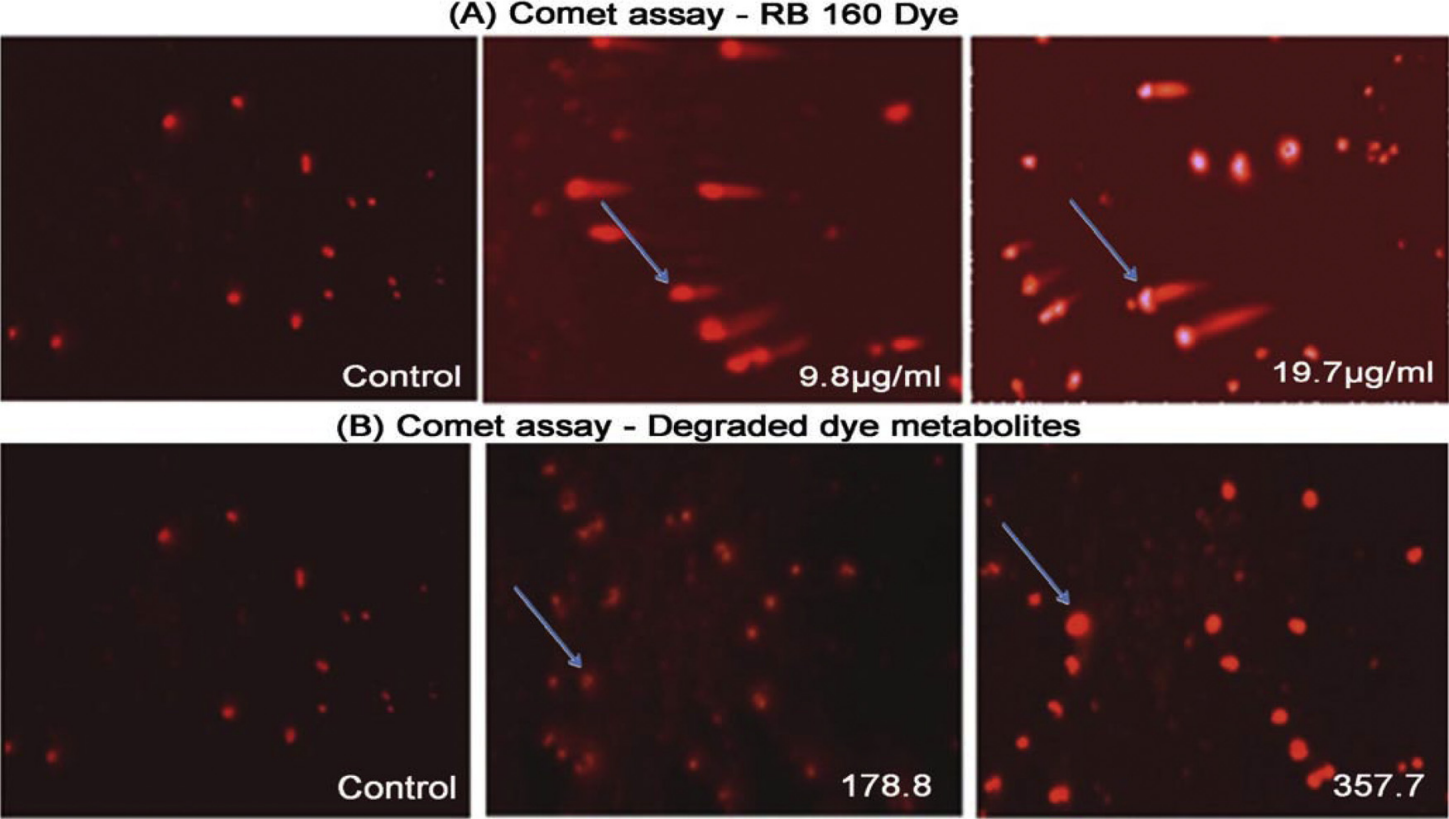
Toxicity analyses using human skin cell line CRL-1474 exposed A) RB 160 and its B) biodegraded product.

Comet assay was performed to determine the genotoxicity of RB160 dye and its biodegradation products. In this study, normal human skin cells CRL-1474 were exposed to RB160 and its degradation products based on the IC_50_ values (19.77 ± 0.46 and 357.7 ± 1.6 μg/ml). Key parameters like percentage of tail DNA (% of DNA in comet tail), tail length (lm) and tail moment were measured and compared with the control. ANOVA test affirmed an important variation between the two groups. The normal cells were exposed to RB160 and showed higher DNA damage than untreated cells (water alone treated cells). Whereas, the degraded products treated cells showed less DNA damage, which is matched to cells of control treatment (Fig. 2). Comet assay was performed to predict the rate of DNA break persuaded by the RB160 dye. The results showed that control cells appeared normal electrophoretic pattern of DNA. However, the native form of RB160 dye exposed cells showed oxidative damaged DNA (26.41 ± 0.24 %) as evidenced by increase in tail length (9.33 ± 0.07 %) and tail moment (1.44 ± 0.07 %). On the other hand, the degraded product showed significantly reduced DNA damage on CRL-1474 (Table 2). These results suggested that degraded dye seems to reduce genotoxic effect on CRL-1474 normal cells. The genotoxic as well as mutagenic impacts seen in RB 160 dye in this study are in coherence with the previous reports, which likewise exhibit the adverse impacts of these types of dyes. Wollin and Gorlitz [38] has been used Ames, MN assay and measuring comet length (in keratinocytes, HaCat line) to observe more than nine dyes (commercial dyes) which had hints of azo dyes, which revealed that these dyes exhibit genotoxic effect on cells *in vitro.*

**Table 2:** Detection DNA damage, Comet tail length and Tail Moment of human skin cells CRL-1474 exposed to RB 160 and degraded products using the Comet assay.

Treatments	Concentrations	DNA in comet tail (%)	Comet tail length (μm)	Tail Moment
Control	–	11.83 ± 0.03^cd^	1.07 ± 0.03^cd^	0.08 ± 0.04^c^
Reactive Blue160 dye (μg/ml)	9.8	19.15 ± 0.04^b^	7.51 ± 0.20^b^	1.18 ± 0.03^ab^
	19.7	26.41 ± 0.24^a^	9.33 ± 0.07^a^	1.44 ± 0.07^a^
Degraded product (μg/ml)	178.8	12.12 ± 0.00^cd^	2.66 ± 0.06^c^	0.91 ± 0.03^b^
	357.7	14.67 ± 0.23^c^	2.33 ± 0.17^c^	0.22 ± 0.01^b^

**Note:** Results are expressed as the means three replicates ± SE. Mean values followed by different letters are significantly different according to the Tukey test at *P* <  0.05.

### 3.5 Effect of RB160 and its degraded products on zebrafish embryonic development

**Fig. 3:**
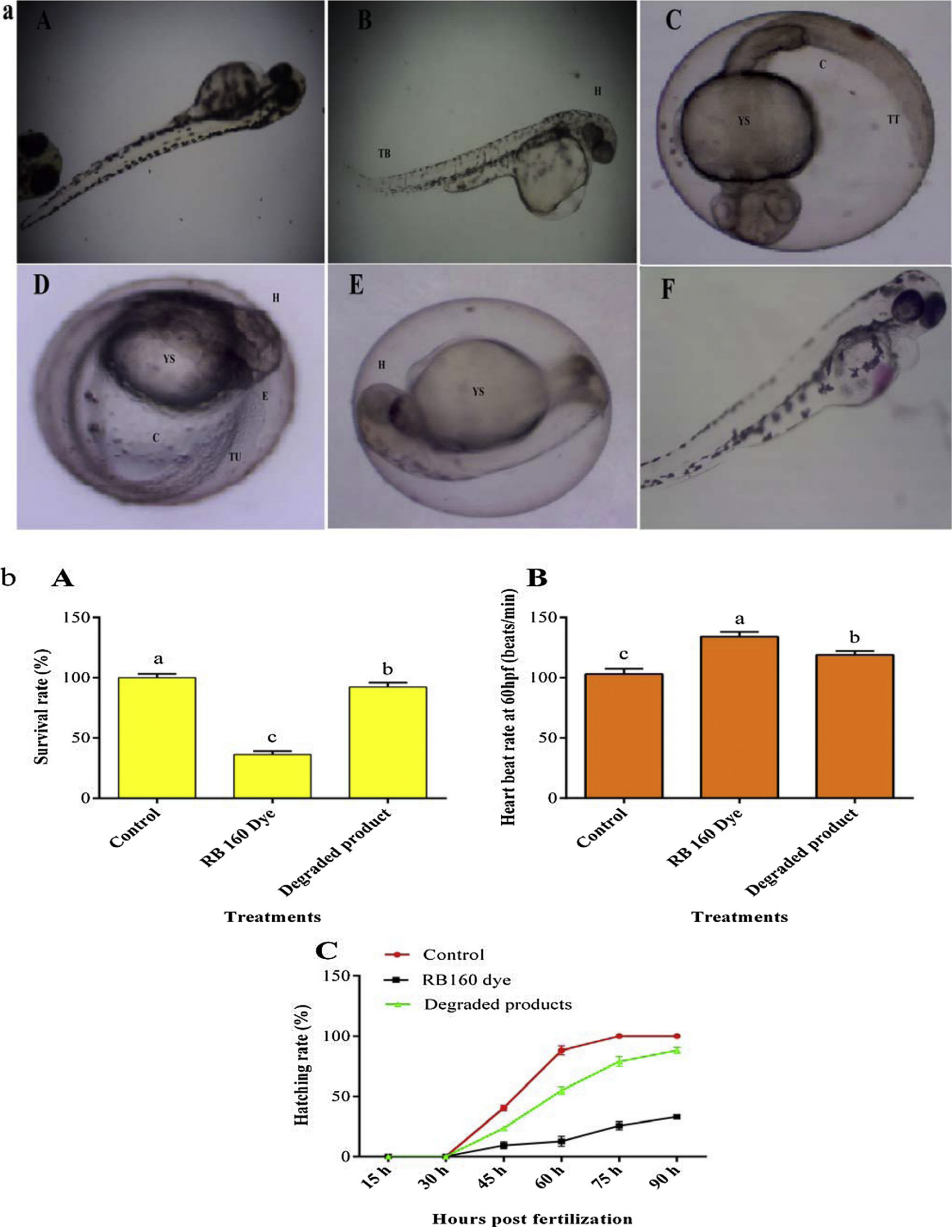
a) Effect of RB 160 dye and degraded product on phenotypic alterations in zebra fish embryos at 96 hpf. A) Control embryo, B) to E) RB 160 dye exposed embryos, F) Degraded product. Yolk sac edema - YS, Tail bend - TB, Eye defect – E, head - H, Curvature - C, Tail ulceration - TU, Tail tip – TT and (b) Effect of RB160 dye and degraded product on survival (A) heart rate (B) and (C) hatching of zebrafish embryos.

In order to confirm the toxic nature of the bacterial reduced product, toxicity study was conducted with zebrafish embryo models with various parameters like phenotypic deformation, survival, hatching and heartbeat rate. Among the treatments, RB160 treated embryos showed several developmental deformities such as tail bend (TB), yolk sac edema (YS), eye defect (E), head (H), curvature (C), tail ulceration (TU), and tail tip (TT) (Fig. 3a). However, control and reduced product treated embryos appeared normally without any phenotypic abnormalities. Recently, Rocha OP and De Oliveira [39] observed developmental abnormalities from tannery effluent treated embryos. Similarly, Shen also observed the similar kind of developmental deformities in Basic Violet 14, Direct Red 28 and Acid Red 26 treated zebrafish embryos.

The maximum hatching and survival rate (100 %, respectively) were obtained in a control embryo group. Correspondingly, reduced product treated embryos showed considerable hatching and survival rate with and 92.13 and 88.31 % respectively. However, the least percentage of hatching and survival rate was observed in RB160 treated embryo groups (Fig. 3b). This might be due to abridged expression of hatching specific enzymes and embryonic movements caused by RB160, which consequently make embryo to fail in the break the non edible outer element of the egg envelope. Waraporn et al. [40] has previously reported that higher concentrations (more than 500 mg/l) of RR239 industrial dye significantly reduce the hatching and the survival rate of zebrafish embryos.

The rate of heartbeat was utilized as a marker in surveying the cardiovascular capacity of zebrafish [41]. It was recorded at 96hpf to find the effects of RB 160 dye and degraded products on the embryos of zebrafish. The control had 110 ± 1.93 beats/min as the average heartbeat, in the case of treated embryos, heartbeat increased (134 ± 2.65 beats/min) significantly. This increased heart beat rate may be due to the association of thiols and metal in the mitochondrial membrane and the construction of free radicals through oxidative phosphorylation and the electron transport chain. Textile dyes have the capability to modulate the cardiovascular function, including cardiac edema, decreased heart rate and decreased blood flow of zebrafish embryo. Interestingly, the rate was reduced (119 ± 1.02 beats/min) in the degraded product treated embryos. These results clearly highlighted that bacterial strain successfully convert toxic RB160 into the less toxic end product.

## 4 Conclusion

The present study proved the competence of bacterial *B. firmus* to degrade and detoxify the industrial reactive dye RB160, and displayed their potential uses in degradation of textile dye and converts non toxic products to the environment. The phytotxicity experiments demonstrated a superior detoxification of RB160 by this strain. This study was conclude the cytotoxicity and genotoxicity of the intact dye and degraded products of dye demonstrate that single bacterial community interceded degradation prompts to the development of nontoxic intermediates affirmed by MTT, comet, and non-target of zebrafish embryo toxicity assays. The competence of the bacterial strain to degrade high concentrations of reactive dyes and convert them into nontoxic by-products for plants, human and aquatic life forms attracts *B. firmus* strain as a potential organism for bioremediation of textile dyes.

## Author statement

The reviewers mentioned changes are carefully corrected by author.

## Declaration of Competing Interest

The authors declare there is no conflict of interest.

## References

[1] Khan Sana, Malik Abdul (2018). Toxicity evaluation of textile effluents and role of native soil bacterium in biodegradation of a textile dye. Environ. Sci. Pollut. Res. - Int..

[2] Panda U.C., Sundaray S.K., Rath P., Nayak B.B., Bhatta D. (2006). Application of factor and cluster analysis for characterization of river and estuarine water system-a case study: Mahanadi River (India). J. Hydrol..

[3] Vimonses V., Jin B., Chow C.W., Saint C. (2010). An adsorption photocatalysis hybrid process using multi-functional nanoporous materials for wastewater reclamation. Water Res..

[4] Ozcan A.S., Erdem B., Ozcan A. (2004). Adsorption of Acid Blue 193 from aqueous solutions onto Na-bentonite and DTMA-bentonite. J. Colloid Interface Sci..

[5] Jonstrup M., Kumar N., Murto M., Mattiasson B. (2011). Sequential anaerobic– aerobic treatment of azo dyes: decolourisation and amine degradability. Desalination.

[6] Meng X., Liu G., Zhou J., Fu Q.S., Wang G. (2012). Azo dye decolorization by *Shewanellaaquimarina* under saline conditions. Bioresour. Technol..

[7] De Aragao U.G., Freeman H.S., Warren S.H., de Oliveira D.P., Terao Y., Watanabe T., Claxton L.D. (2005). The contribution of azo dyes to the mutagenic activity of the Cristais River. Chemosphere.

[8] Bose Rajan Babu, Thillaichidambarama Muneeswaran, Paulraj Balaji, Narayanan Kalyanaraman, Ganesan Nandhagopal, Muthiaha Ramakritinan Chokalingam, Murugesan Rajesh Kannan (2018). Bio-decolourization of Reactive Blue EFAF using halotolerant Exiguobacterium profundumstrain CMR2 isolated from salt pan. Biocatal. Agric. Biotechnol..

[9] Spagni A., Casu S., Crispino N.A., Farina R., Mattioli D. (2010). Filterability in a submerged anaerobic membrane bioreactor. Desalination.

[10] Baban A., Yediler G., Avaz S.S., Hostede (2010). Biological and oxidative treatment of cotton textile dye-bath effluents by fixed and fluidized bed reactors. Bioresour. Technol..

[11] Wasti Ayesha, Ali Awan M. (2016). Adsorption of textile dye onto modified immobilized activated alumina. J. Assoc. Arab. Univ. Basic Appl. Sci..

[12] Santhanama Manikandan, Selvaraj Rajeswari, Veerasubbian Vinothkumar, Sundaram Maruthamuthu (2019). Bacterial degradation of electrochemically oxidized textile effluent: performance of oxic, anoxic and hybrid oxic-anoxic consortium. Chem. Eng. J..

[13] Srinivasan Shantkriti, Sadasivam Senthil Kumar, Gunalan Seshan, Shanmugam Gnanendra, Kothandan Gugan (2019). Application of docking and active site analysis for enzyme linked biodegradation of textile dyes. Environ. Pollut..

[14] Kolekar M.Y., Nemade N.H., Markad L.V., Adav S.S., Patole S.M., Kodam M.K. (2012). Decolorization and biodegradation of azo dye, reactive blue 59 by aerobic granules. Bioresour. Technol..

[15] Harrelkas F., Paulo A., Alves M.M., El. Khadir L., Zahraa O., Pons M.N., van der Zee F.P. (2008). Photocatalytic and combined anaerobicphotocatalytic treatment of textile dyes. Chemosphere.

[16] Zainal Z., Hui L.K., Hussein M.Z., Taufiq-Yap Y.H., Abdullah A.H., Ramli I. (2005). Removal of dyesusing immobilized titanium dioxide illuminated by fluorescent lamps. J. Hazard. Mater..

[17] Asad S., Amoozegar M.A., Pourbabaee A.A., Sarbolouki M.N., Dastgheib S.M. (2007). Decolorization of textile azo dyes by newly isolated halophilic and halotolerant bacteria. Bioresour. Technol..

[18] Moosvi, Keharia H., Madamwar D. (2005). Decolorization of textile dye Reactive Violet by a newly isolated bacterial consortium RVM 11.1. World J. Microbiol. Biotechnol..

[19] Balapure H. Kshama, Jain Kunal, Chattaraj Sananda, Nikhil Bhatt S., Madamwar Datta (2014). Co-metabolic degradation of diazo dye—reactive blue 160 by enriched mixed cultures BDN. J. Hazard. Mater..

[20] Taskin M., Erdal S. (2010). Reactive dye bioaccumulation by fungus Aspergillus niger isolated from the effluent of sugar fabric-contaminated soil. Toxicol. Ind. Health.

[21] Taskin M., Erdal S. (2010). Reactive dye bioaccumulation by fungus Aspergillus niger isolated from the effluent of sugar fabric-contaminated soil. Toxicol. Ind. Health.

[22] Jadhav J.P., Kalyani D.C., Telke A.A., Phugare S.S., Govindwar S.P. (2010). Evaluation of the efficacy of a bacterial consortium for the removal of color, reduction of heavy metals, and toxicity from textile dye effluent. Bioresour. Technol..

[23] Wu Lihua, Yi Huilan, Yi Min (2010). Assessment of arsenic toxicity using Allium/Vicia root tip micronucleus assays. J. Hazard. Mater..

[24] Ma T.H., Grant W.F., de Serres F.J. (1997). The genotoxicity monitoring of the air, water and soil a preliminary report of the International Program on Plant Bioassays IPPB. Mutat. Res..

[25] Rajaguru P. (1997). Studies on Some Aspects of Tirupur Environment and the Use of Soil Bacteria in the Degradation of Azo Dyes.

[26] Rajeswari K., Subashkumar R., Vijayaraman K. (2014). Degradation of textile dyes by isolated *Lysinibacillus Sphaericus* strain RSV-1 and *Stenotrophomonas maltophilia* strain RSV-2 and toxicity assessment of degraded product. J. Environ. Anal. Toxicol..

[27] Kurade M.B., Waghmode T.R., Kagalkar A.N., Govindwar S.P. (2012). Decolorization of textile industry effluent containing disperse dye Scarlet RR by a newly developed bacterial-yeast consortium BL-GG. Chem. Eng. J..

[28] Kalyani D.C., Telke A.A., Dhanve R.S., Jadhav J.P. (2009). Ecofriendly biodegradation and detoxification of Reactive Red 2 textile dye by newly isolated *Pseudomonas* sp. SUK1. J. Hazard. Mater..

[29] Ayed Lamia, Kouidhi Bochra, Bekir Karima, Bakhrouf Amina (2013). Biodegradation of azo and triphenylmethanes dyes: cytotoxicity of dyes, slime production and enzymatic activities of Staphylococcus epidermidis isolated from industrial wastewater. Afr. J. Microb. Res..

[30] Saghirzadeh M., Gharaati M.R., Mohammadi S., Ghiassi N.M. (2008). Evaluation of DNA damage in the root cells of *Allium cepa* seeds growing in soil of high background radiation areas of Ramsar, Iran. J. Environ. Radioact..

[31] Geetha A., Palanisamy Pn., Sivakumar P., Ganesh Kumar P., Sujatha M. (2008). Assessment of underground water contamination and effect of textile effluents on noyyal river basin in and around tiruppur town, Tamil Nadu. E-J. Chem..

[32] Parshetti G., Kalme S., Saratale G., Govindwar S. (2006). Biodegradation of malachite green by *Kocuria rosea* MTCC 1532. Acta Chim. Slov..

[33] Phugare S. Swapnil, Kalyani C. Dayanand, Patil Asmita V., Jadhav Jyoti P. (2011). Textile dye degradation by bacterial consortium and subsequent toxicological analysis of dye and dye metabolites using cytotoxicity, genotoxicity and oxidative stress studies. J. Hazard. Mater..

[34] Carita R., Marin M.M.A. (2008). Induction of chromosome aberrations in the *Allium cepa* test system caused by the exposure of seeds to industrial effluents contaminated with azo dyes. Chemosphere.

[35] Chakraborty R., Mukherjee A.K., Mukherjee A. (2009). Evaluation of genotoxicity of coal fly ash in *Allium cepa* root cells by combining comet assay with the Allium test. Environ. Monit. Assess..

[36] Bafana A., Chakrabarti T., Devi S.S. (2008). Azoreductase and dye detoxification activities of Bacillus velezensis strain AB. Appl. Microbiol. Biotechnol..

[37] Chetan C., Oturkar M.S., Patole K.R., Gawai D. (2013). Madamwar Enzyme based cleavage strategy of *Bacillus lentus* BI377 in response to metabolism of azoic recalcitrant. Bioresour. Technol..

[38] Wollin K.M., Gorlitz B.D. (2004). Comparison of genotoxicity of textile dyestuffs in the Salmonella mutagenicity assay, *in vitro* micronucleus assay, and single cell gel/comet assay. J. Environ. Pathol. Toxicol. Oncol..

[39] Rocha O.P., de oliveira D.P. (2017). Investigation of a Brazilian tannery effluent by means of zebra fish (Danio rerio) embryo acute toxicity (FET) test. J. Toxicol. Environ. Health Part A.

[40] Jungtanasombut Waraporn, Preeprem Pichapop, Kovitvadhi Satit, Kovitvadhi Uthaiwan, Hannongbua Supa (2014). Effects of Reactive Red 239 on developing zebra fish (Danio rerio) embryos. Nat. Sci..

[41] Shen Bing, Liu Hong-Cui, Ou Wen-Bin, Eilers Grant, Zhou Sheng-Mei, Meng Fan-Guo, Li Chun-Qi, Li Yong-Quan (2015). Toxicity induced by basic violet 14, direct red 28 and acid red 26 in Zebrafish larvae. J. Appl. Toxicol..

